# New Drug and Biologics Approvals in 2019: A Systematic Analysis of Patient Experience Data in FDA Drug Approval Packages and Product Labels

**DOI:** 10.1007/s43441-020-00244-x

**Published:** 2020-11-23

**Authors:** Katrine Schultz-Knudsen, Ugne Sabaliauskaite, Johan Hellsten, Anders Blaedel Lassen, Anne Vinther Morant

**Affiliations:** 1 Regulatory Science & Strategy, H. Lundbeck A/S, Valby, Denmark; 2 Regulatory Dossier Management, H. Lundbeck A/S, Valby, Denmark; 3 Patient Insights, H. Lundbeck A/S, Valby, Denmark; 4 Anne Morant Consulting, Frederiksberg, Denmark

**Keywords:** Patient experience data, FDA, Patient-focused drug development, Label claim, Regulatory decision-making, Patient-reported outcomes

## Abstract

**Background:**

The FDA Patient-Focused Drug Development Initiative was launched to ensure the incorporation of the patient voice into drug development and evaluation. Since 2017, the FDA must publish a statement outlining patient experience data (PED) considered in the approval of new drugs. This study investigated the presence and role of PED in drug approval and translation into product label claims.

**Methods:**

PED reported in approval packages of the 48 drugs approved by FDA’s Center for Drug Evaluation and Research in 2019 was identified and categorized. PED in the form of clinical outcome assessments (COAs) was characterized by endpoint positioning and outcome. The product labels were analyzed for PED-related claims.

**Results:**

PED was reported as relevant for 39 of 48 (81.3%) drugs approved in 2019. COAs were the predominant PED type; other PED was identified for only 9 (18.8%) drugs, and none included qualitative or patient preference studies. COAs were the only type of PED for which associated claims were identified in the product labels. 27 out of 48 (56.3%) labels contained one or more efficacy claims based on COAs; of these, patient-reported outcomes were the most prevalent with claims identified in 19 labels (39.6%).

**Conclusion:**

There are ample opportunities for incorporating PED beyond COAs to inform drug development and facilitate availability of medicines tailored to patient needs. A higher level of transparency on the role of PED in regulatory decision-making and a clear path to PED-based label claims could incentivize sponsors and enable patient empowerment in treatment decisions.

**Electronic supplementary material:**

The online version of this article (10.1007/s43441-020-00244-x) contains supplementary material, which is available to authorized users.

## Introduction

In recent years, patient contributions to the regulatory eco-system have evolved from what was primarily a process scope to also play a central role in evidence generation. Leading regulatory agencies have embarked on different initiatives aimed at strengthening the patient relevance in drug development and regulatory decision-making. Examples include the US Food and Drug Administration’s (FDA) Patient-Focused Drug Development (PFDD) program [1]; the European Medicines Agency’s Regulatory Science Strategy to 2025 [2]; and Japan’s Pharmaceutical and Medical Devices Agency’s ‘Patients First’ initiative [3].

In 2012, under the fifth authorization of the Prescription Drug User Fee Act, FDA initiated a series of PFDD meetings to obtain the patient perspective on specific diseases and experiences with available treatments [4]. The 21st Century Cures Act signed into law in 2016 gave further momentum to FDA’s PFDD efforts, with FDA developing a series of four methodological guidance documents. These address how stakeholders can collect and submit patient experience data (PED) and other relevant information from patients and caregivers for drug development and regulatory decision-making [5, 6].

‘Patient experience data’ includes data that is collected by any individuals (patients, parents, caregivers, patient advocacy organizations, researchers, research sponsors, or other parties) and is intended to provide information about patients’ experiences with a disease or condition [5]. According to the FDA PFDD Glossary [7], PED can be interpreted as “information that captures patients’ experiences, perspectives, needs, and priorities related to (but not limited to):

1. the symptoms of their condition and its natural history;
2. the impact of the conditions on their functioning and quality of life;
3. their experience with treatments;
4. input on which outcomes are important to them;
5. patient preferences for outcomes and treatments; and
6. the relative importance of any issue as defined by patients.”

Under Section 3001 of the 21st Century Cures Act, for drug applications submitted after June 2017, the FDA is required to publish a brief statement regarding the PED and related information submitted and reviewed as part of the application [8]. The FDA fulfills this requirement by including a table of “Patient Experience Data Relevant to this Application” (hereafter referred to as the ‘PED table’) in their publicly available reviews of new drug and biologics license applications. The PED table captures the following categories of PED: (1) PED that was submitted by the sponsor as part of the application and considered relevant by the FDA, including clinical outcome assessments (COAs) divided into four subcategories (patient-, clinician-, and observer-reported outcomes as well as performance outcomes); qualitative studies; PFDD or other stakeholder meeting summary reports; observational survey studies; natural history and patient preference studies; or ‘other’, and (2) PED not submitted as part of the application but considered relevant by the FDA in the review. This includes input informed from participation in meetings with patient stakeholders; PFDD or other stakeholder meeting summary reports; observational survey studies; or ‘other’. As such the PED table captures a range of PED categories that may relate broadly to one or more of the types of PED as interpreted in the PFDD Glossary and outlined above.

In a recent publication, FDA drug approvals from 2018 were reviewed with focus on the FDA’s reporting of PED in the PED table. The most prominent source of PED was patient-reported outcomes (PROs) [9]. PROs can provide information from patients about the symptoms of their disease, functioning or quality of life, or in some cases even patient experience or preference with the treatment. However, as the individual PRO typically focuses on only one of these dimensions, and as patient interviews or other methods may be needed to capture dimensions such as patient preference for treatments and outcomes, it does not necessarily reflect the patient experience with the broader set of objectives that can be informed by PED as per the list above.

As the PFDD Initiative is still in its infancy, the value and the role played by PED in context of drug development and FDA approval remain somewhat unclear. To obtain a better understanding of this, the present analysis investigated PED included in New Drug Applications and Biologics License Applications approved by the FDA’s Center for Drug Evaluation and Research’s (CDER) in 2019. Information on PED was collected not only from the FDA PED table but also as reported elsewhere in the FDA Review. In addition, the study included an analysis of the extent to which PED identified in the FDA Reviews translates into the United States Prescribing Information (hereafter referred to as the label), ideally allowing for conveying the patients’ experience with the treatment to healthcare professionals and to the patients themselves.

## Methods

### Drugs Approved by FDA in 2019: FDA Reviews and Product Labels

All New Molecular Entities including original biologics (hereafter collectively referred to as NMEs) approved by the FDA’s CDER from 1 January 2019 through 31 December 2019 were identified via the FDA New Drug Therapy Approvals overview [10]. The associated FDA approval packages (hereafter referred to as the ‘FDA Review’) and original product labels were retrieved from the Drugs@FDA website [11]. The NMEs were grouped into therapeutic areas according to the American Hospital Formulary Service Pharmacologic-Therapeutic Classification system [12, 13]. All FDA Reviews and product labels were assessed by two independent authors; discrepancies were assessed independently by a third author and resolved by consensus.

### Patient Experience Data as Reported in the FDA PED Table

Each FDA Review was assessed for inclusion of a PED table as well as type of PED reported in the PED table (please refer to the introduction for the list of individual PED types captured in the PED table). In cases where the PED table was replaced by the sentence “PED was not submitted as part of this application” a PED table was assessed as present.

### Patient Experience Data as Identified Elsewhere in the FDA Reviews

To identify PED belonging to the categories reported in the PED table as well as additional PED that might have been submitted and/or considered in the review, the FDA Reviews were further analyzed to identify and classify such data. This included data falling within the overall types of PED as categorized in the PED table, including both COAs and other types of PED such as PFDD meeting reports, qualitative studies, etc.

### Clinical Outcome Assessments as a Source of Patient Experience Data

As the PED table identifies COAs as a source of PED, COAs were included in the analysis if they formed the basis of a clinical study endpoint. Thus, the analysis excluded COAs applied solely as a basis for inclusion criteria, baseline assessment, or regular safety assessments such as, e.g., physical examination or assessments of suicidal ideation which is standard for psychiatric and selected non-psychiatric drugs [14]. Clinical outcome assessments were categorized by type in accordance with the PED table categories and as defined by the BEST (Biomarkers, EndpointS, and other Tools) Resource [15]: (patient-reported outcome [PRO], clinician-reported outcome [ClinRO], observer-reported outcome [ObsRO], or performance outcome [PerfO]). Furthermore, COAs were categorized according to endpoint positioning (primary, secondary, or exploratory) as well as outcome (positive, negative, or interpretation unclear as per the FDA assessment; primary and secondary endpoints only). COAs that formed the basis of safety endpoints exclusively (included in the pivotal studies or in dedicated safety studies, e.g., abuse potential studies) were categorized as safety endpoints regardless of endpoint positioning. In cases where a single COA formed the basis of more than one study endpoint, the COA was categorized by the highest endpoint status (i.e., primary if both primary and secondary). COAs that informed both efficacy and safety were classified by the efficacy endpoint status only. COAs that were composed of items from two different categories (e.g., containing both patient- and clinician-reported items) were counted in both categories.

When COAs were not explicitly defined by category in the FDA Review, classification into PRO, ClinRO, ObsRO, and PerfO was performed based on the information provided in the FDA Review and according to the definitions provided in the FDA PFDD glossary [7]. If only limited information was available in the FDA Review, the FDA COA Compendium [16] and/or published literature were consulted for classification.

### Assessment of Patient Experience Data Translated into Product Label Claims

For assessment of the communication of potential PED in the product label, the labels were systematically analyzed for mention of each PED identified in the FDA Reviews. This comprised both PED reported in the PED table and any additional PED identified by the authors (including COAs as outlined above) in the FDA Reviews.

## Results

### Patient Experience Data Included in FDA Reviews

**Figure 1.**
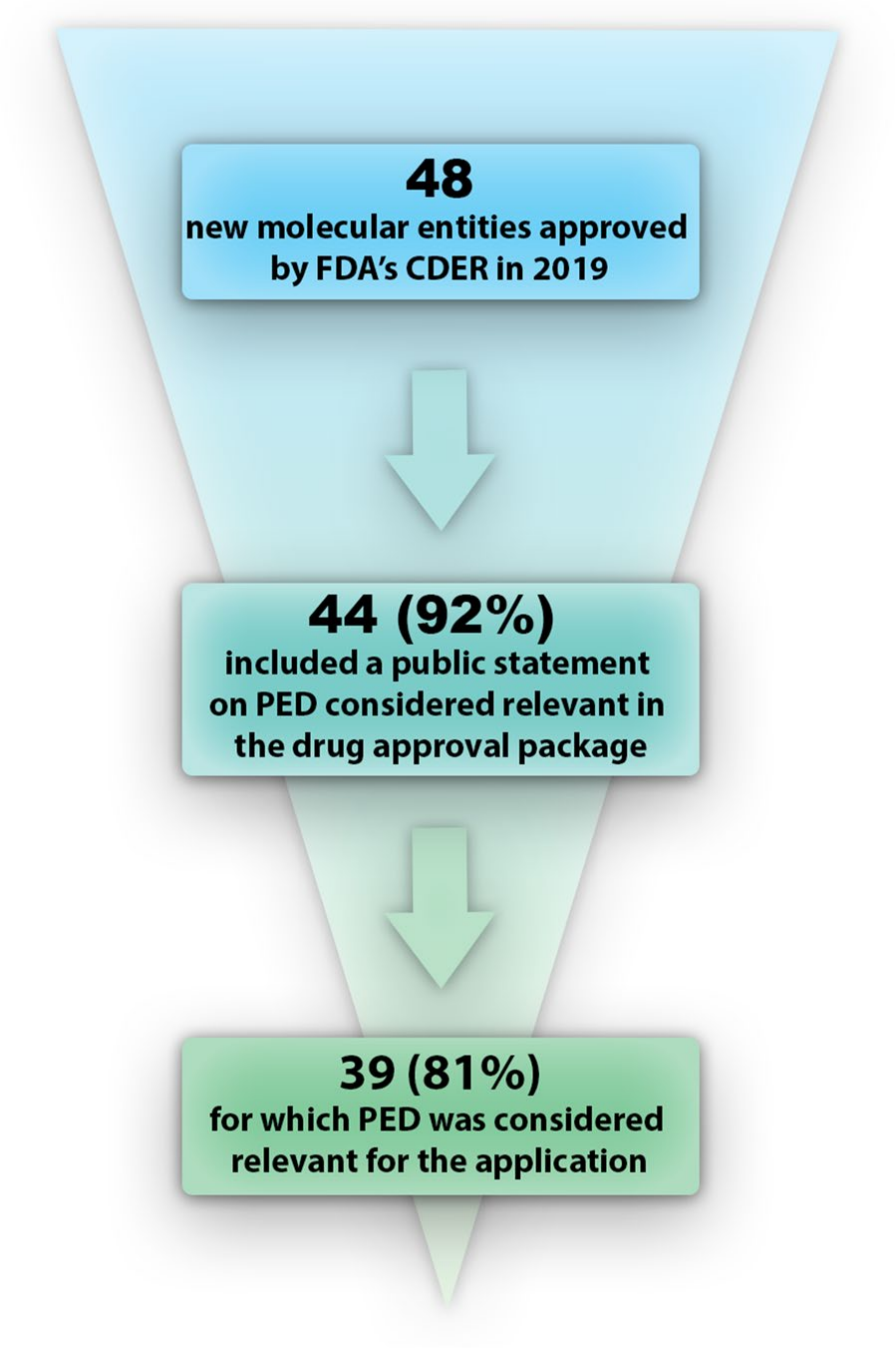
Overview of the Number of New Molecular Entities Approved by the FDA Center for Drug Evaluation and Research in 2019 and Inclusion of Patient Experience Data in the Application as Reported by the FDA.

In 2019, 48 NMEs including four diagnostic agents were approved by FDA’s CDER. A completed PED table (or a statement that no PED was submitted as part of the application) was included in 44 of the 48 FDA Reviews (91.7% compared to ~ 80% in 2018) [9]. According to the information reported in the PED table, PED relevant to the application was submitted by the sponsor and/or taken into consideration by the FDA during the review for 39 of those 44 NMEs (88.6% compared to 70.8% in 2018 [9]; Fig. 1).

**Figure 2.**
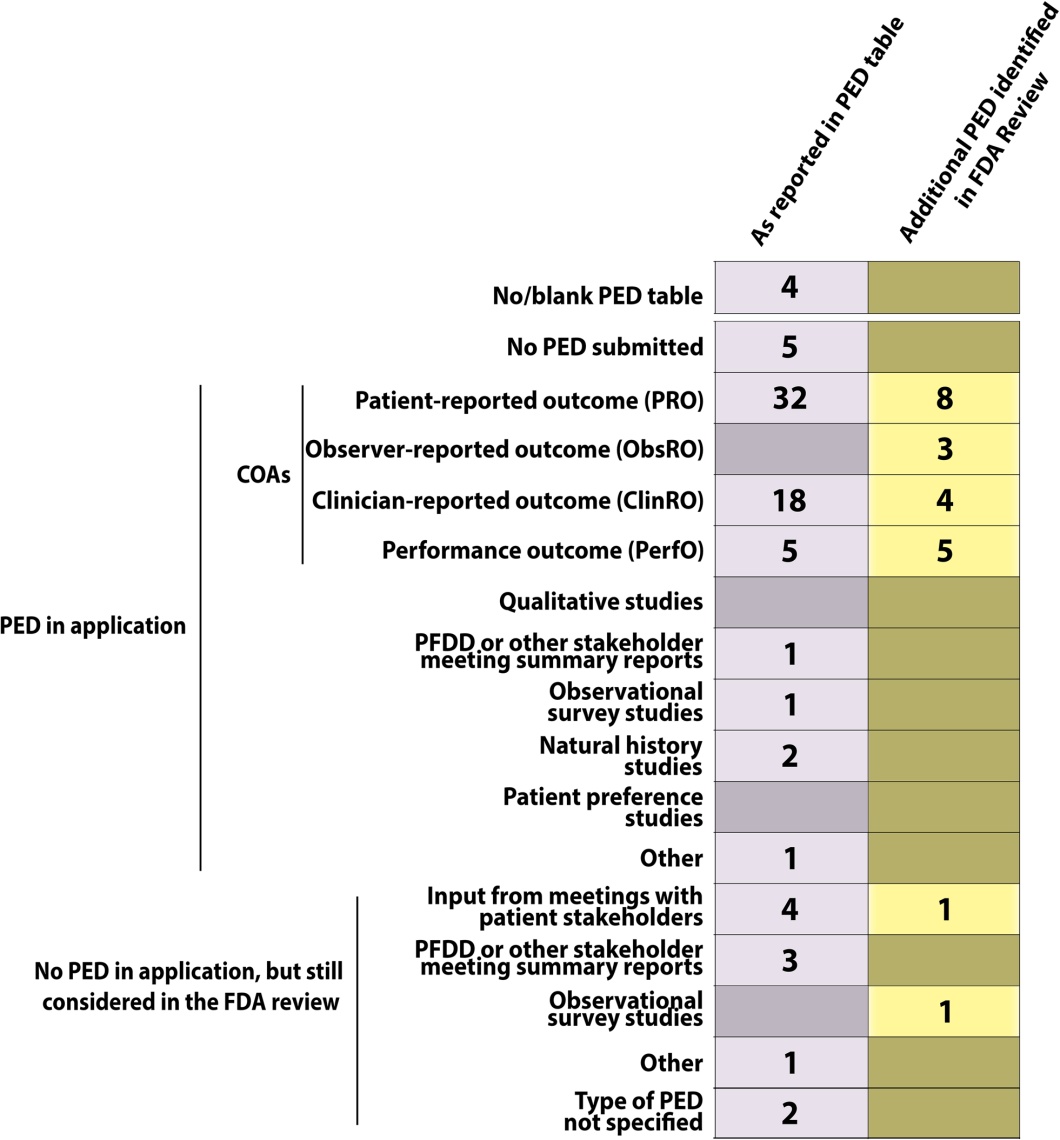
Summary of Types of Patient Experience Data (PED) Submitted and/or Considered by the FDA as Part of the Applications for Marketing Authorization for Drugs Approved by the FDA’s Center for Drug Evaluation and Research in 2019. The FDA Reviews published for four of the approved drugs either did not include a PED table, or the PED table was left blank. The first (gray) column denotes the number of drugs that included the individual type of PED as reported in the PED tables. The second (yellow/brown) column shows the number of drugs for which any additional PED was identified by the authors elsewhere in the FDA Review and not reported by type in the PED table.

In line with the previously published analysis of FDA-reported PED in FDA approvals from 2018 [9], COAs constituted by far the most common source of PED (Fig. 2). In fact, 38 of the 39 NMEs, for which PED was reported as relevant for the application, included PED in the form of one or more types of COAs. The only exception was fam-trastuzumab deruxtecan for which the FDA considered PED (type not specified) that was not submitted by the sponsor to be relevant for the application (Fig. S1).

Patient-reported outcomes were reported as PED relevant to 32 out of 48 (66.7%) applications. ClinROs, PerfOs and ObsROs were less often reported [18 (37.5%), 5 (10.4%), and 0 (0%) applications, respectively]. Additional COA categories beyond those reported in the PED tables as relevant to the application were identified by the authors in eight out of 48 (16.7%) FDA Reviews in the case of PROs. ClinROs, PerfOs, and ObsROs were identified in additionally 4 (8.3%), 5 (10.4%), and 3 (6.3%) FDA Reviews, respectively (Fig. 2). In these FDA Reviews, either no PED table was included, or the COA category was not reported in the PED table. Lastly, ClinROs could not be identified in three FDA Reviews in which ClinROs were checked as PED relevant to the application (Fig. S1).

One or more types of PED other than COAs were reported in the PED table as relevant to the application for only 9 of 48 (18.8%) NMEs. These consisted of PFDD or other stakeholder meeting summary reports (4 applications); input informed from participation in meetings with patient stakeholders (4 applications); observational survey studies (1 application) and natural history studies (2 applications). Notably, PED in the form of qualitative studies or patient preference studies was reported as relevant to zero applications (Fig. 2).

In general, inconsistency in terms of how the PED tables were completed was observed. In several cases a specific PED category was checked off but could not be readily identified in the FDA Review, or PED was identified and clearly discussed in the FDA Review as pertinent to the application, but not checked off in the PED table (Fig. S1).

### Patient Experience Data Included in Product Labels

An assessment of PED-related label claims showed that only PED in the form of COAs resulted in label claims in this cohort. For 27 of the 48 NMEs (56.3%), one or more COA-based efficacy claims were included in the originally approved product labels. The labels of 19 drugs (39.6%) included one or more PRO-based efficacy claims; 11 (22.9%) had efficacy claims based on ClinROs; three (6.3%) based on PerfOs and one (2.1%) included an efficacy claim based on an ObsRO (Fig. S2; Table S1).

**Figure 3.**
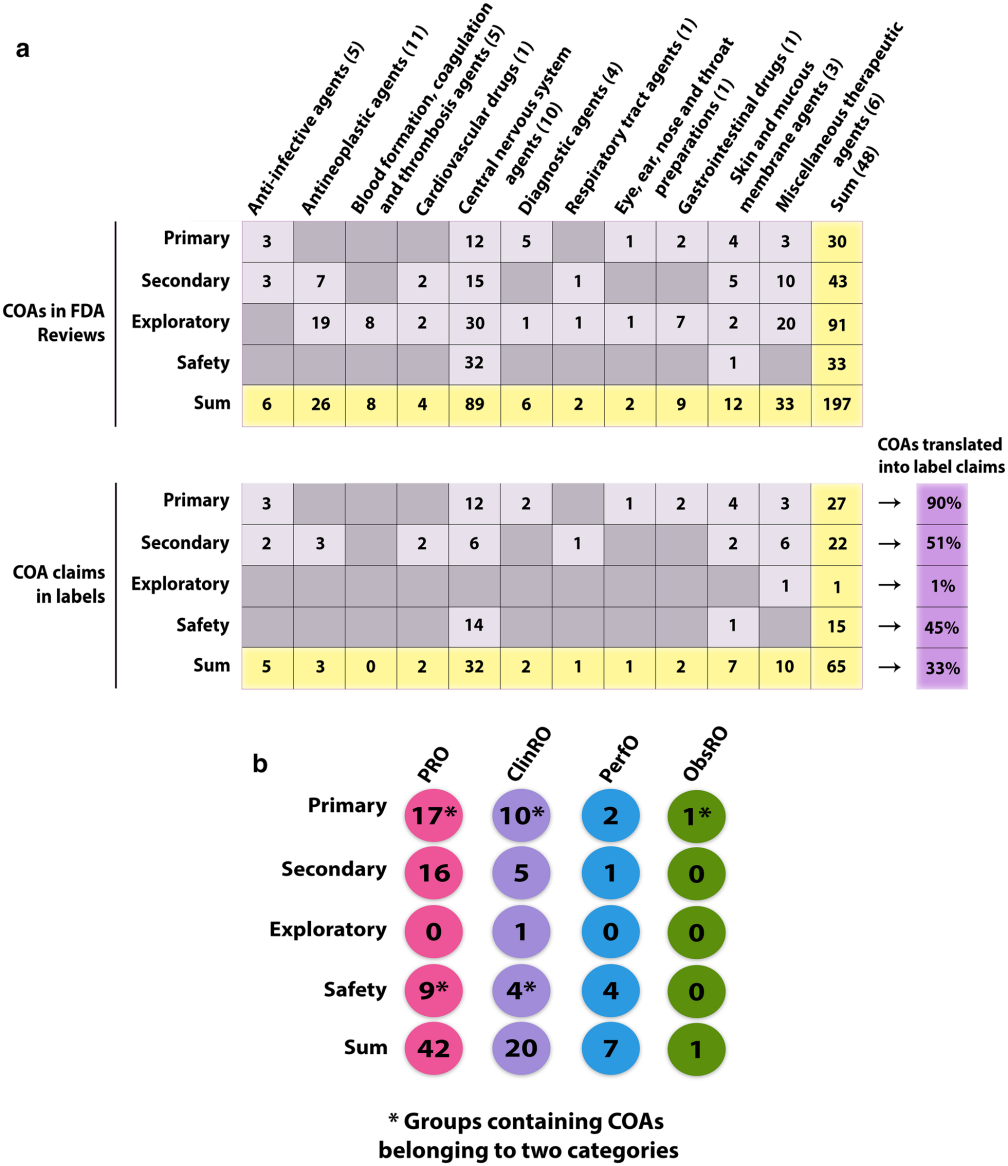
Overview of Clinical Outcomes Assessments (COAs) Identified in FDA Reviews and Labels of New Molecular Entities Approved by the FDA Center for Drug Evaluation and Research in 2019. **a** Number of individual COAs forming the basis of label claims divided by therapeutic area and endpoint positioning. Numbers in parentheses denote the amount of drugs approved within the given therapeutic area. The proportion of COAs identified in the FDA Reviews and resulting in a label claim is denoted in purple for each endpoint positioning. **b** Number of COA-based label claims divided by COA category (PRO, ClinRO, PerfO, ObsRO) and endpoint positioning. *Note that the numbers do not add up with the numbers in **a** due to COAs composed of items falling within two different categories (e.g., COAs composed of both PRO and ClinRO items) being counted twice in this illustration.

All except one of the COA efficacy claims were based on primary or secondary efficacy endpoints (Fig. 3a, b) for which a convincing and statistically significant (when relevant) effect had generally been demonstrated (Table S1). The one exception was siponimod for which a secondary ClinRO endpoint included in the label was considered exploratory due to endpoints higher in the testing hierarchy failing to reach statistical significance [17]. Negative label claims, i.e., mention of no effect demonstrated on a specific efficacy endpoint as observed for bremelanotide, constitute another exception (Table S1).

In terms of the total amount of COAs that resulted in efficacy-related label claims, 33 out of 53 (62.3%) were PROs, 16 (30.2%) were ClinROs, and three (5.7%) and one (1.9%) were PerfOs and ObsROs, respectively (Fig. 3b).

One or more safety-related claims, i.e., a statement that the drug did not appear to be associated with a deterioration as measured by specific safety-related COAs, were identified in six (12.5%) product labels (Fig. S2; Table S1). 14 out of a total of 15 (93.3%) safety-related label claims were for drugs to treat central nervous system disorders (Fig. 3a).

### Clinical Outcome Assessments and Therapeutic Area Variation

Therapeutic areas differed both in the number of different COA categories, as well as in the absolute number of individual COAs included in the clinical development programs (Figs. 3a, S2). Clinical trials supporting the approval of oncology drugs (antineoplastic agents) typically included a limited number of COAs, and mainly as exploratory PRO endpoints. In only 3 out of 11 oncology drugs did a PRO (darolutamide and fedratinib) or a ClinRO (pexidartinib) result in a label claim (Figs. 3a, S2). Likewise, COAs were scarcely used in clinical trials supporting approval of ‘blood formation, coagulation and thrombosis’ drugs, and only as exploratory endpoints (Fig. 3a).

In contrast, COAs were prominent in central nervous system clinical development programs. These typically comprised two or more different COA categories and often formed the basis of both primary, secondary, exploratory, and safety endpoints (Figs. 3a, S2).

## Discussion

### Does Patient Experience Data Adequately Reflect Patient Experience?

While the FDA PED table captures a diverse range of PED categories, the PFDD Glossary interpretation of PED appears even broader [7]. With this in mind, the scope of the PED included in drug applications approved by the FDA in 2019 appears very narrow as it primarily constitutes COAs (Fig. 2). In fact, it could be argued that as COAs have been included in the clinical development programs as basis for trial endpoints for decades, their inclusion adds little new in the context of PED unless explicitly added for this purpose.

The observation that none of the applications included qualitative or patient preference studies may appear surprising in light of the focus placed by FDA and other stakeholders—including the pharmaceutical industry—on patient centricity in drug development. It could be speculated that qualitative research involving patients may be conducted for example in the clinical design phase to inform endpoint strategies, and by commercial functions for market research purposes, yet not submitted as part of the application. If the value of such data in the regulatory benefit/risk assessment was clearer, sponsors might take the necessary steps in terms of study design and Independent Ethics Committee approval to leverage the data also in the regulatory context. We would therefore argue that there is an underutilized opportunity for sponsors to think PED beyond COAs and beyond the traditional clinical trial context.

### Do Clinical Outcome Assessments Per Definition Represent Patient Experience Data?

As per the FDA PED table, PROs, ClinROs, ObsROs and PerfOs all constitute PED; yet, it is unclear if all COAs are per definition regarded as representing PED, or whether it comes down to an individual assessment of each COA. For the sake of this study, all COAs forming the basis of efficacy endpoints were included. COAs that formed the basis of endpoints in dedicated safety studies were included, whereas COAs that were incorporated for regular safety assessments only (e.g., physical examination or assessments of suicidal ideation) were excluded. Depending on whether the sponsor and/or the FDA had noted such assessments as PED relevant to the application, this might explain the cases in which ClinROs checked in the PED table could not be identified in the FDA Review. On the other hand, as the COAs considered relevant for the application are only specified by type and not by the specific instrument in the majority of the PED tables this may lead to an overestimation in this study of the number of COAs that represent PED. As such, a definition of when COAs may represent PED is needed, and a clear identification in the PED table of the individual COAs considered relevant for the application would be desirable to improve the transparency in the regulatory decision-making.

In the FDA Reviews, the COAs are primarily evaluated in their capacity as a basis for clinical trial endpoints and rarely discussed in context of clinical relevance of the COA-detected drug effect for the patient (Table S1). This may lead to the perception that there is a disconnect between COAs and actual patient-relevant outcomes.

Lastly, it might be argued that PROs are the only type of COAs to actually report the patient experience as the remaining COA types are rated by clinicians or observers such as caregivers. Even if PROs may capture diverse aspects of treatment outcomes, these would still represent a limited part of the broader spectrum of patient experience data as outlined in the introduction. For example, in only three cases were PROs that specifically captured treatment satisfaction identified (Table S1); as was the case for the majority of the PROs, all three formed the basis of exploratory endpoints (Fig. S2) that were not further discussed in the FDA Reviews. One reason so many PRO endpoints are included as exploratory only might be that they are included primarily for non-regulatory purposes such as to support market access. Yet, their exploratory status means that potentially important and patient-relevant information is not communicated to the patients, as exploratory endpoints are rarely included in the FDA product label (Fig. 3a, b).

### Clinical Outcome Assessments and Therapeutic Area Variation

Almost 40% of NMEs approved by the FDA’s CDER in 2019 included one or more PRO-based label claims. Keeping in mind the small sample size and lack of comparison of proportion of drugs falling within so-called ‘PRO-dependent’ therapeutic areas, this suggests a remarkable increase compared to the proportion of FDA-approved drugs with PRO label claims reported in 2011–2015 (16.5%) and 2006–2010 (24.1%) [18, 19]. The authors of these earlier studies attributed the difference between these two periods to a substantial increase in 2011–2015 of NMEs approved within oncology and other therapeutic areas which do not typically rely on PRO data [19]. An observed reason in the present analysis for the low proportion of PRO-based label claims within oncology is that the FDA typically did not evaluate submitted PRO data in context of benefit/risk with reference to challenges with interpretation of PRO data from uncontrolled trials (Table S1), as also previously reported [20].

In line with the previous observations pertaining to PRO-based label claims, this therapeutic area variation is also clearly reflected in the present analysis, both for PROs specifically and for COAs in general, and both in terms of use in clinical trials and translation into label claims. In PRO-dependent disease areas such as central nervous system disorders, where biomarkers and objective clinical outcomes are scarce, and where dedicated safety trials are often required (e.g., abuse potential or sedation studies), the role of COAs is threefold as COAs often form the basis of both primary endpoints, product differentiation (secondary or exploratory endpoints), and safety. Yet, COAs and PROs in particular may add important information and provide context for the primary efficacy results in terms of clinical meaningfulness of the treatment, regardless of therapeutic area.

### The Role of Patient Experience Data in the FDA Benefit/Risk Assessment: Need for Transparency

In a few cases, one or more COAs were discussed in the FDA Reviews but were not reported in the PED table. For example, the ubrogepant FDA Review [21] specifies that no PED was submitted as part of the application; yet the co-primary endpoints (pain freedom and absence of most bothersome symptom) are based on patients’ self-report [22] and as such would constitute PROs. As these were co-primary endpoints in the pivotal trials, it could be argued that they should be characterized as PED relevant for the ubrogepant application. Another example is darolutamide for which the ‘Regulatory Background’ section outlines FDA’s direct engagement with the patient community to explore difficulties with drug development in the indicated population. This engagement led to publication of a draft guidance and provided justification for the choice of the primary endpoint for the clinical development program. Yet, the corresponding category is not checked in the PED table [23].

In some instances, the discrepancy between the PED reported as relevant to the application in the PED table and the PED identified in the FDA Reviews as part of this analysis may reside in the wording ‘relevant to the application’; i.e., the PED may not have been considered relevant in the context of the overall benefit/risk assessment. Even so, the threshold for when PED should be checked off in the PED table as deemed relevant for the application is unclear based on the present sample and current FDA guidance.

In other instances, the PED that was reported in the PED table could not be readily identified in the FDA Reviews. While this was the case for a modest proportion of the reported COAs, it seemed to be almost the rule for non-COA PED such as PFDD or other stakeholder meeting reports or natural history studies. As such, the role of non-COA PED in regulatory decision-making remains particularly obscure. For sponsors to invest in PED such as qualitative research and cope with the added complexity related to developing such data to satisfy regulatory standards, there needs to be a clearly perceived added value.

To illustrate a welcomed level of transparency to contextualize the value of the FDA PFDD Program for drug development and approval, selected case examples are listed in Table 1. Such clear identification of the source of PED in the FDA Reviews as well as a consistent reporting on the impact (if any) of the specific PED on the regulatory decision-making could help sponsors improve the basis for drug approval. Understanding the added value of integrating PED into all stages of clinical development could potentially serve as an incentive for sponsors and eventually result in the development of more clinically meaningful treatments.

**Table 1.** Examples of Clear Identification and Discussion of Patient Experience Data in FDA Reviews.

Desirable Level of Transparency	Case Examples
Clear identification of individual COAs in PED table	The solriamfetol and brexanolone FDA Reviews identified the specific COAs evaluated by the FDA as relevant to the application in the PED table [29, 30]
Clear identification of specific PFDD meeting report	The crizanlizumab PED table not only checked “PFDD or other stakeholder meeting summary reports” but also provided a hyperlink to clearly identify the specific meeting report [31]
Discussion of clinical relevance and impact of PRO endpoint results	In the alpelisib FDA Review, the FDA discussed the results of an exploratory PRO endpoint and did not agree to the sponsor’s conclusion that the changes in global health status/Quality of Life were not clinically meaningful. The FDA concluded that “it is not possible to exclude a detrimental effect of alpelisib on global health status or quality of life for patients” [32]
Rationale for recommendation of COA-based claims for inclusion in the label	In the upadacitinib FDA Review, the FDA reviewer discussed the rationale for recommending inclusion of each individual COA endpoint in the label. Although patient experience is not explicitly mentioned, the individual COAs and associated endpoints are discussed in the context of clinical relevance [33]
Thorough discussion of individual COAs and choice of primary endpoint based on PFDD meeting	The bremelanotide FDA Review carefully outlines the properties of each COA and also describes how the outcome of an FDA-led PFDD meeting and scientific workshop with patients and practitioners resulted in the sponsor amending the endpoint hierarchy to reflect the relevance of the individual endpoints to the patient population in question [34]

### Communication of Patient Experience Data to Patients and Prescribers

The single most important vehicle for communication of the efficacy and safety of any drug is the product label. None of the few cases of non-COA PED reported in the FDA Reviews translated into mentions in the label. This is expected as these data were typically not specific to the drug but general for the patient population (e.g., PFDD reports). In case of COAs, the reasons for any non-acceptance for label claims were not systematically discussed in the FDA Reviews. Apart from sponsors not applying for label claims, reasons for non-acceptance (when discussed) included difficulty in data interpretation due, e.g., to missing data or single arm study design. In general, exploratory endpoints were simply not considered in the context of the label.

While there seems to be an increase in the proportion of drugs with PRO-based label claims in 2019 compared to 2006 through 2015 [18, 19], none of the NMEs approved by FDA in 2019 had non-COA PED such as qualitative studies or patient preference studies included in the label, let alone the applications. Moreover, in those labels that included COA-based claims, it was not clearly stated in the label which claims represent actual patient experience. As such, PED cannot be not readily identified nor interpreted by the prescribers and the patients.

One of the lessons learned from the numerous FDA-led disease-specific PFDD meetings is that “patients want their experience described with the words that they use to best describe how it feels” [24]. A greater focus on inclusion of PED in the label as well as a description of what these data mean to the patient would provide a two-way communication of incorporating the patient’s voice in drug development and approval.

The 2017 FDA approval of a sub-cutaneous formulation of the originally intravenously administered rituximab is a great case example to illustrate inclusion of well-described PED in the label: The sponsor conducted a patient preference study demonstrating that the sub-cutaneous formulation was preferred by the patients mostly due to administration requiring less time in the clinic. These results were included in a dedicated Section 14.4 ‘Patient Experience Data’ in the label supporting not only prescribing decisions but also allowing the sponsor to advertize that patients themselves prefer the new formulation [25]. This new, dedicated section of the product label offers an opportunity for sponsors to develop and communicate PED beyond COAs to patients and prescribers.

The public statement on PED considered relevant for the application as materialized in the PED table is a modest step in the right direction toward disclosing the efforts invested by sponsors and the FDA into incorporating the patient voice in drug development and approval. An easily accessible, sponsor-authored and FDA-endorsed lay-language narrative on the inclusion and role of PED in drug development could not only serve as a valuable source of information for patients, caregivers and prescribers but also provide more transparency and a better understanding of the role of PED in regulatory decision-making.

### Study Limitations

There are some limitations to this study in addition to those already discussed: first, the study is based on a small sample size of 48 NMEs. The investigation included FDA approvals from 2019 only, as the legislative requirement for FDA to make public a brief statement on PED is applicable to applications submitted after June 2017 [8], and because an analysis of FDA-reported use of PED has been published for drugs approved in 2018, albeit with a different scope [9]. Second, as the clinical development programs were likely designed several years before the year of approval (allowing time for conduct and reporting of the studies as well as submission, review and approval of the application for marketing authorization), the PED included in 2019 drug approvals does not reflect the 2019 drug development standard, let alone the current guidance. Third, the study focused on FDA specifically, as the FDA has developed a well-structured framework to promote incorporating the patient voice in drug development. Fourth, as information about rejected applications is not systematically available [26], the study looked only at approved drugs and as such would not capture any potential rejections related to (the lack of) PED. Lastly, no patient- or patient organization representatives were involved in the conduct of this study; statements of what might be desirable from the patient perspective should be interpreted with this is mind.

### Next Steps in Patient-Centric Drug Development

To date, the FDA has invested significant efforts into fostering patient engagement in drug development by creating a well-structured PFDD framework including establishing a platform for multi-stakeholder meetings with patient representatives (PFDD meetings), as well as a series of guidance documents [1, 24]. The FDA draft guidance on Developing and Submitting Proposed Draft Guidance Relating to Patient Experience Data [27] is another example of FDA’s efforts to connect patient experience with drug development by encouraging the patient communities to share what is important to them and what could be relevant PED for informing drug development and approval. However, based on the present analysis one could argue that there is still room for improvements across stakeholders.

The PED table provides a starting point toward a more structured use and reporting of PED in drug development and approval. However, real impact will need more transparency on the data itself and its use in regulatory decision-making. This could be done by building further projects like the FDA’s Project Patient Voice [28], aiming at communicating oncology PRO data that are usually not included in the product label. This could be expanded to meaningfully cover a broader set of PED across therapeutic areas.

Sponsors have the responsibility to incorporate PED in drug development in order to ensure that the resulting treatments are meaningful to the patients; yet a better understanding of how PED may facilitate drug development and evaluation is needed. Under the 21st Century Cures Act Title III, Section 3004, the FDA is required to publish by June 1 of 2021, 2026, and 2031 “a report assessing the use of patient experience data in regulatory decision-making, in particular with respect to the review of patient experience data and information on patient-focused drug development tools as part of applications.” These reports will hopefully help clarify the role of PED in regulatory decision-making. This in turn could potentially elevate the importance of PED to a level that extends beyond relying on the COAs that would in most cases be included in the clinical trials to support approval, regardless of their categorization as PED.

## Conclusions

The majority (81.3%) of the NMEs that were approved by the FDA CDER in 2019 included PED as reported in the FDA PED tables. Various discrepancies were observed in reporting of PED, and the most common type of PED, namely COAs, was rarely discussed in the context of patient experience. Beyond COAs, other types of PED such as reports from FDA PFDD meetings or input informed from other meetings with patients, natural history- or observational studies, were included or considered in only a few applications. Notably, two of the PED categories that may have the greatest potential for representing the patient voice in drug development, namely qualitative studies, such as patient or caregiver interviews, and patient preference studies, were not reported at all.

The analysis of PED-based label claims revealed that COAs were the only type of PED described in the label; while one or more COA-based claims of efficacy were included in more than half of the approved labels, these were neither identified, nor described in the context of patient experience.

Even though the FDA’s PFDD framework is still relatively new, this analysis points to a range of missed opportunities for including PED beyond COAs in recent drug development efforts and not least in the label.

Drug development and review tools supporting a higher level of transparency in terms of the role of PED in regulatory decision-making would be welcomed to enable sponsors to better understand—and plan for—the importance of PED in drug development and approval. A greater focus from both the FDA and the sponsors on clearly identifying PED, explaining what it means to the patients, and translating the PED into label claims would fulfill a dual purpose of creating incentives for sponsors to expand generation of PED while ensuring that the patient experience is communicated in a way that can enhance patient, caregiver, and healthcare professional empowerment in treatment decisions.

## Electronic supplementary material

Below is the link to the electronic supplementary material.

(PNG 5616 kb) **Supplementary Fig. 1** Detailed overview of patient experience data (PED) reported by the FDA as relevant to the applications for marketing authorization for new molecular entities approved by the FDA Center for Drug Evaluation and Research in 2019. Checked circles indicate PED checked in the PED table. Grey background shading means the particular type of PED was identified in the FDA Review as part of this analysis. No background shading means PED could not be identified in the FDA Review. The far right column designates compounds for which the authors identified potential PED that was not reported by FDA in the PED table

(PNG 2946 kb) **Supplementary Fig. 2** Absolute number of clinical outcome assessments (COAs) identified in drug approval packages and labels of new molecular entities approved by the FDA Center for Drug Evaluation and Research in 2019 as illustrated for individual drugs and grouped by therapeutic area. Each circle represents an individual COA; a filled circle signifies the inclusion of a claim based on the COA in the label. Each COA is classified by the highest endpoint positioning as described in the methods (primary > secondary > exploratory > safety only). Pink = Patient Reported Outcome (PRO); purple = Clinician Reported Outcome (ClinRO); blue = Performance Outcome measure (PerfO); green = Observer Rated Outcome (ObsRO). Circles composed on two colors signify that the COA is composed of items from both categories. The – in the PRO-based secondary endpoint of bremelanotide signifies that the endpoint resulted in a ‘negative’ label claim stating that no difference to placebo was demonstrated on this endpoint

(DOCX 54 kb)
