# Supplementary material for: New Drug and Biologics Approvals in 2019: A Systematic Analysis of Patient Experience Data in FDA Drug Approval Packages and Product Labels
Source: Ther Innov Regul Sci. 2020 Nov 23;55(3):503–13. doi: 10.1007/s43441-020-00244-x (PMC8021513; doi:10.1007/s43441-020-00244-x)
Supplement: Supplementary file 3 — (DOCX 54 kb) [file 43441_2020_244_MOESM3_ESM.docx]

# Supplementary Table 1: Complete overview of clinical outcome assessments (COA) identified in the FDA Review and labels of new molecular entities approved by the FDA Center for Drug Evaluation and Research in 2019. COA categories that were checked in the patient experience data (PED) table by FDA as relevant for the application but not readily identified in the FDA Review are emphasized in *italics*, as are COAs that were identified in the FDA Review as part of the present analysis but not reported by category in the PED table.

| Drug | Summary of FDA-approved use on approval date | COAs identified in FDA Review | COAs included in label | Additional remarks |
| --- | --- | --- | --- | --- |
| afamelanotide | Increase pain-free light exposure in patients with erythropoietic  protoporphyria | PROs: Likert pain-intensity scale (1+); EPP-QoL (2+); DLQI (2÷) | Pain free light exposure (based on Likert pain-intensity scale) | The Applicant has not submitted information to assess whether the QoL tools are fit for purpose and they may not be clinically meaningful 🡪 results not considered in the overall assessment |
| air polymer-  type A | Diagnostic agent for fallopian tube assessment | PRO: VAS pain score (E)  *ClinRO: none identified* | - | Approval based on published studies  As per the PED table, ClinRO data was submitted, however not identified in the FDA Review |
| alpelisib | Advanced or metastatic breast cancer | PROs: EORTC QLQ-C30 (E); EQ-5D-5L (E); BPI-SF (E)  *ClinRO: EGOC-PS (E)* | - | PRO analyses not controlled for multiple comparisons, and therefore considered exploratory  The review of the PRO results did not identify a large decrement in symptoms or function that would materially alter the overall favorable B/R determination |
| bremelanotide | Hypoactive sexual desire disorder in premenopausal women | PROs: FSFI-D Q1&2 (1+); FSDS-DAO Q13 (1+); number of SSEs as measured by FSEP-R Q10 (2÷); EDQ (E); GAQ (E); WITS-9 (E); BSS (S); various “Drug liking” measures (S) | FSFI-D Q1&2; FSDS-DAO Q13;  No sign. difference in no. of SSEs | No rationale provided for including label statement on lack of difference in number of SSEs.  EDQ and GAQ “*provided supportive data but were not sufficiently robust to support labeling claims*”  The pivotal studies included a treatment satisfaction PRO (Women’s inventory of treatment satisfaction; WITS-9), but this PRO is not discussed in the FDA Review |
| brexanolone | Postpartum depression | PROs: EPDS (E); GAD-7 (E); PHQ-9 (E); BIMF (E); SF-36 (E); HCRU (E); StSS (S); “Drug Liking” scores (S)  ClinROs: HAM-D (1+); CGI-I (E) | HAM-D  Safety: Various “Drug Liking” measures | “*The Applicant is seeking labeling claim based on CGI-I*”  No rationale included to explain why labeling claim not obtained.  The C-SSRS (ClinRO) was part of the regular safety assessment |
| brilliant  blue G | Dye used in eye surgery | ClinROs: slit-lamp biomicroscopy (1); Surgeon’s Intraope-rative Assessment of Facility of Dye Usage (1)  *PerfO: BCVA/VA (1)* | - | 505b2 application based on literature only. The approval was supported by 12 published studies  Limited description of individual endpoints (primary, secondary, or exploratory) in FDA Review  No clinical studies described in the label. |
| brolucizumab | Wet age-related macular degeneration | *PRO: VFQ-25 (E)*  PerfO: ETDRS (1+) | ETDRS | PED table only specifies that a PerfO was included |
| caplacizumab | Acquired thrombotic thrombocytopenic purpura | - | - | No PED submitted as part of application as stated in PED table |
| cefiderocol | Complicated urinary tract infection | PRO: Subject Reported Symptoms as part of composite response (microbiological eradication and clinical cure; (1+) and clinical response (2+) | Composite response; clinical response | No specific discussion of subject reported symptoms in the context of PED |
| cenobamate | Partial-onset seizures | *PRO: Various “Drug Liking” measures (S)*  *PerfOs: CRT (S); DAT (S); SSTM task (S)*  *ObsRO: OAA/S (S)* | *Safety: Various “Drug liking” measures* | No PED table included in FDA review  The human abuse potential study included an extensive list of secondary outcome measures related to “Drug Liking” + cognitive/ psychomotor impairment and observer-rated measures of sedation  The C-SSRS (ClinRO) was part of the regular safety assessment  No PED was identified beyond the abuse study. Seizure frequency was reported via a patient diary; however not deemed PRO as the report included no assessment of the severity by the patient (only presence or absence) |
| crizanlizumab | Reduce vaso-occlusive crises in sickle cell disease | PRO: BPI-Q (2→E); SF-36 (E) | - | Primary endpoint not statistically significant → BPI-Q exploratory endpoint → not included in the USPI  “*The PRO endpoints were exploratory and thus were not included in the prescribing information*” |
| darolutamide | Non-metastatic prostate cancer | PROs: BPI-SF (2+), EQ-5D-3L (E), FACT-P (E), EORTC-QLQ-PR25 (E)  *ClinRO: ECOG-PS (E)* | Delay in time to pain progression (BPI-SF) | PRO analyses exploratory as they were not included in the testing hierarchy and Type I error was not controlled for multiple comparisons  *Considering that it is difficult to differentiate whether pain is related to cancer or not, the size of effect, sensitivity analysis results (…) improvement in time to pain progression will be included descriptively in the label* |
| elexacaftor/  ivacaftor/  tezacaftor | Cystic Fibrosis | PROs: CFQ-R RD (2+); TSMQ (E) | CFQ-R RD | No specific discussion of CFQ-R RD in context of labeling  TSMQ not reviewed in detail |
| enfortumab  vedotin | Refractory bladder cancer | PROs: EORTC-QLQ-C30 (E); EQ-5D (E)  *ClinRO: none identified* | - | In a single arm study, the PRO data were analyzed descriptively only, and no statistical inference can be made  Applicant concluded that the PRO data support the efficacy and safety results and demonstrate that QoL was maintained  ClinRO checked in PED table, but no ClinROs identified that formed the basis of an endpoint (ECOG-PS and slit lamp eye examination were regular safety assessments only) |
| entrectinib | Metastatic non-small cell lung cancer and locally advanced or metastatic solid tumors with a specific genetic defect | PROs: QLQ-C30 (E); QLQ-LC13 (E); EQ-5D (E) | - | In a single arm trial, the PRO results are not interpretable. Only a descriptive analysis of PRO outcomes was conducted, and all the PRO analysis results were considered exploratory  ECOG-PS and Lansky or Karnofsky performance status (ClinROs) were part of the regular safety monitoring |
| erdafitinib | Locally advanced or metastatic bladder cancer | PROs: SMQ (E); GIC-S (E); GIC-QoL (E) | - | The SMQ “*is limited in its ability to ascertain the cause of any decreased level of functioning or QoL for an individual patient”*  “*The collection of patient-reported outcomes for this NDA was too rudimentary to allow an in-depth analysis*” |
| fam-trastuzumab deruxtecan | Metastatic breast cancer | - | - | PED table reported no PED submitted as part of the application, however PED (not specified) was considered in this review. |
| fedratinib | Certain types of myelofibrosis | PROs: MFSAF (2+); EQ-5D (E); MPN-SAF (E) | MFSAF | Results for individual symptom scores of MFSAF included in FDA Review  EQ-5D and MPN-SAF only mentioned in FDA Summary Review and not discussed in Multidisciplinary Review → concluded exploratory  ECOG-PS (ClinRO) was part of the safety assessment, but not clearly defined as the basis of an endpoint |
| ferric maltol | Iron deficiency anemia | - | - | No PED submitted as part of application as stated in PED table |
| fluorodopa  F 18 | Diagnostic agent for Parkinsonian syndromes | *ClinRO: Readers’ independent evaluation of PET images (positive and negative % agreement) (1+)* | *Readers’ independent evaluation of PET images (positive and negative % agreement)* | No PED table included in FDA Review  Doubt as to whether the outcome measure should be categorized as COA resolved by consensus and COA specialist advice; conclusion was ClinRO rather than biomarker as the outcome is based on the clinicians’ interpretation (ClinRO) of the PET images (biomarker) |
| Ga-68-  DOTATOC | Diagnostic agent for neuroendocrine tumors | *ClinRO: Rating of images by two independent readers 🡪 positive & negative % agreement (1+)* | *Rating of images by two independent readers 🡪 positive &* *negative % agreement* | No PED submitted as part of application as stated in PED table  Doubt as to whether the outcome measure should be categorized as COA resolved by consensus and COA specialist advice; conclusion was ClinRO rather than biomarker as the outcome is based on the clinicians’ interpretation (ClinRO) of the Ga-68-DOTATOC images (biomarker) |
| givosiran | Acute hepatic porphyria | PROs: BPI-SF (2÷); SF-12 PCS (E); EQ-5D-5L (E); days missed of work/school (E); PGIC (E), PPEQ (E) | - | Given that worst pain scores (based on BPI-SF) failed statistical significance, all subsequent secondary endpoints (including SF-12 PCS) were viewed as exploratory endpoints  PROs/QoL measures not discussed further in FDA Review |
| golodirsen | Duchenne muscular dystrophy | PerfOs: 6MWT (2÷); NSAA (E); PUL (E); Timed 4-step test (E); MoviPlate (E); Pinch and Hand Grip (E)  *PRO: PODCI* (E) | - | “*Both 6MWT and FVC measures decreased from baseline and there was no correlation observed between dystrophin levels* [primary surrogate endpoint] *and 6MWT measures*”  Exploratory endpoints only mentioned as ‘other endpoints’ with no results reported  6MWT was secondary outcome measure but not accounted for in the SAP |
| imipenem/  cilastatin/  relebactam | Complicated urinary tract infections and complicated  intra-abdominal infections | ClinRO: Clinician ascertained clinical response (1/2 → E) | - | *“Two studies not appropriately designed for hypothesis testing (no reliable conclusions to be concluded based on results); one study descriptive only with no hypothesis testing”*  Efficacy primarily established by in vitro and animal studies and supported by previous findings for the antimicrobial components |
| istradefylline | Parkinson’s disease “off” episodes | PROs: Patient diary OFF time (1+*) and ON time (2+); PGI-I (2÷); PDQ (2÷); SF-35 (2÷)  ClinROs: UPDRS (2÷); CGI (2÷) | OFF time;  ON time without troublesome dyskinesia | Initial application resulted in a non-approvable letter (in 2008); clinical reviewer recommended against approval of the present re-submitted NDA  In almost all the studies, there was no adjustment for multiplicity for secondary endpoints 🡪 nominal p-values only  The C-SSRS (ClinRO) was part of the regular safety assessment |
| lasmiditan | Migraine with or without aura | PROs: eDiary-based assessment of pain (1+) and MBS (2+); PGIC (E); Interference in daily activity (E); “Drug Liking” scores (S); S-PSD (S); KSS (S); VAS-MSAD (S)  *PerfOs: SDLP (S); CogScreen SDC (S); various Collision & Speed measures (S)* | Freedom from pain and MBS  Safety: *SDLP;* “Drug Liking” scores | FDA reviewer comment on ‘Interference in daily activity’ measure: *While PROs are important in assessing the benefit of a drug, this measure is not a validated one, and was only an exploratory endpoint. I would not recommend including this information in the label.*  PerfOs not checked in PED table, and arguably not included in the pivotal studies; however PerfOs were included in the driving performance studies and reported (SDLP) in the label  The C-SSRS (ClinRO) was part of the regular safety assessment |
| lefamulin | Community-acquired bacterial pneumonia | PRO: ECR (1+)  ClinRO: IACR (2+) | ECR, IACR | The primary endpoint (Early clinical response; ECR) was a composite of non-COA clinical outcomes (survival and receipt of non-study antibacterial treatment) and PROs (patient reported assessment of symptoms severity) |
| lemborexant | Insomnia | PROs: sSOL (1+); sWASO (2+); sSEF (2+); sTST (2*); ISI (E); FSS (E); PGI-I (E); WPAI-GH (E); EQ-5D-3L (E); KSS (S); T-BWSQ (S); eC-SSRS (S); diverse “Drug liking” measures (S)  PerfOs: PAB (S); SDLP (S); DSST (S); PVT (S); RTI (S); MSLT (S); AAT (S) | sSOL, sSEF, sWASO  Safety:  PAB;  SDLP;  T-BWSQ  AAT (ability to awaken to sound)  eC-SSRS (suicidal ideation)  “Drug liking” measures | Prominent role of PerfOs and PROs in special safety studies  “*Results from analyses of primary and key secondary endpoints provide the principal evidence for effectiveness. The studies also provide evidence supporting the clinical meaningfulness of the improvements in sleep parameters; these secondary and exploratory endpoints included objective and patient-reported measures*”  “*Although the change from baseline* [on secondary endpoints] *to Days 1/2 was not a pre-specified endpoint within the statistical hierarchy, this information is considered highly relevant to clinicians and warrants inclusion in labeling with careful language that does not suggest that this was a prespecified endpoint with appropriate type I error control*” |
| lumateperone | Schizophrenia | *PRO: SWN-S (E)*  ClinROs: PANSS (1+*); CGI-S (2*/E); PSP (E); CDSS (E); SAS (S); AIMS (S)  *PRO/ObsRO: PSQI (E)*  *ClinRO/PRO: BARS (S)* | PANSS  Safety: SAS; AIMS; *BARS* | Benefits: *“Results on secondary or exploratory endpoints are not relevant for discussion in this review. There was no prospective plan to control the Type-I error rate for the secondary endpoints. Therefore, all secondary endpoints were regarded as exploratory”*  Risks: The Applicant assessed extrapyramidal symptoms using BARS, AIMS, and SAS … no meaningful changes from baseline  Only ClinROs reported in PED table  The C-SSRS (ClinRO) was part of the regular safety assessment |
| luspatercept | Anemia associated with beta thalassemia | PROs: SF-36 (E); TranQoL (E) | - | “*There were no prespecified statistical hypotheses in regard to comparison of the HRQoL endpoints and study was not powered to detect any differences”* → all analyses are descriptive” |
| pexidartinib | Symptomatic tenosynovial giant cell tumor | PROs: PROMIS Physical Function (2*); Worst Stiffness NRS item (2*); BPI- Worst Pain NRS item (2÷)  ClinRO: ROM (2+) | ROM | “*While the analysis of PROMIS Physical Function and Worst Stiffness NRS item demonstrated a statistically significant improvement, the results may not reliably estimate treatment effects due to a high proportion of missing data and thus, valid inference cannot be made*” |
| pitolisant | Excessive daytime sleepiness in patients with narcolepsy | PROs: ESS (1+); Daily/Weekly Cataplexy Attacks (E); EQ-5D (E); Patient’s global opinion on the effect of the drug (E)  ClinROs: BDI (S); CGI-C (E)  *PerfO: SART (2*)* | ESS | Varying results observed compared to placebo and active compactor using CGI-C, EQ-5D, and Patient’s global opinion on the effect of the drug between the two trials |
| polatuzumab  vedotin | Relapsed or refractory diffuse large B-cell lymphoma | PRO: TINAS (E) | - | PRO results not interpretable due to high proportion of missing data. |
| prabotulinum-  toxin A | Improve appearance of glabellar lines | PROs: GAIS (E), SSS (E)  PRO/ClinRO: GLS (1+) | GLS | “*As the endpoints based on these PROs* [GAIS and SSS] *were designated as ‘exploratory’ and not included in the multiplicity testing strategy, the efficacy results for these endpoints are not presented in this review*” |
| pretomanid | Treatment-resistant forms of tuberculosis | *PRO: EQ-5D (2*)* | - | No PED table in FDA Review.  Resolution of symptoms as measured by Patient Self-Reported Health Status [EQ-5D] was difficult to interpret without a comparator group  ClinROs identified as part of the regular safety assessments (slit lamp eye examination among others), but not as basis for trial endpoints |
| risankizumab | Moderate-to-severe plaque psoriasis | PROs: PSS (2+); DLQI (2+); EuroQoL (E); HADS (E)  ClinROs: sPGA (1+); PASI (1+) | sPGA; PASI; PSS | COA review concludes that PSS is adequate to support a labeling claim related to improvement in psoriasis signs and symptoms  “*The DLQI does not appear to be fit-for-purpose for labeling in the context of this drug development program due to lack of item relevancy*” |
| romosozumab | Osteoporosis | PROs: EQ-5D-5L (E); BPI worst pain (E); OPAQ SV (E); LAD (E); WOMAC (E)  *ClinRO: none identified* | - | No consistent or clinically meaningful differences in PRO/ClinRO endpoints were identified  As per the PED table, ClinRO data was submitted, however not identified in the FDA Review. |
| selinexor | Relapsed or refractory multiple myeloma | PRO: FACT-MM (2*) | - | PRO data only available from small proportion of patients; extremely challenging to interpret PRO data in single arm trial → not evaluated in context of B/R assessment |
| siponimod | Relapsing forms of multiple sclerosis | ClinRO: EDSS (1+); ARR (2→E+)  PerfO: T25FW (2÷)  *PRO: MSWS (2→E)* | EDSS; ARR (w. nominal p-value);  No sign. difference on T25FW | ARR described under ‘other secondary endpoints’; claim included in label with a nominal p-value as key secondary endpoint (T25FW) did not reach statistical significance  MSWS clearly identified as PRO in FDA review even if this category was not checked off in PED table. MSWS is not discussed further in the FDA Review |
| solriamfetol | Excessive daytime sleepiness in patients with narcolepsy or  obstructive sleep apnea | PROs: ESS (1+); PGI-C (2+), FOSQ-10 (E), SF-36v2 Health Survey (E), EuroQol (E), WPAI:SHP (E); cataplectic events (E); Drug Liking Score (S)  ClinRO: CGI-C (2+)  *PerfO: MWT (1+)* | ESS; PGI-C; MWT  Safety: Drug Liking Score | ESS and MWT^[[1]](#footnote-2)^ used as co-primary endpoints in most of the studies, and PGI-C almost consistently used as key secondary; all with convincingly positive results. CGI-C was included in some studies and mostly as secondary endpoint; generally positive results.  Exploratory PROs generally positive across trials  The C-SSRS (ClinRO) was part of the regular safety assessment |
| tafamidis  meglumine | Cardiomyopathy caused by transthyretin-mediated amyloidosis | PROs: KCCQ-OS (2+); PtGA (E); EQ-5D-3L (E)  PerfO: 6MWT (2+) | KCCQ-OS; 6MWT | Study optimally designed to assess all three important outcomes: mortality, function, and QoL.  All subgroups demonstrated a consistent beneficial effect with respect to both the 6MWT and the KCCQ-OS  PtGA and EQ-5D-3L not discussed in FDA Review. |
| tenapanor | Irritable bowel syndrome with constipation | PROs: CSBM (1+/2*/E); abdominal pain (1+/2+/E); straining (E); abdominal bloating (E); discomfort (E); fullness (E); cramping (E); stool consistency (E); IBS-QOL (E) | CSBM; abdominal pain | Both CSBM and abdominal pain were components of the primary endpoint (overall response), and the response on the individual components were key secondary endpoints  Some of the additional, exploratory PRO elements were questioned by the review team and not endorsed by the FDA COA staff; the results on these endpoints were not discussed in the FDA Review |
| triclaben-  dazole | Fascioliasis | *PRO/ObsRO: Clinical symptoms (1/E*)* | *Clinical symptoms* | PED table included in FDA review but left blank  Clinical efficacy supported partially by literature, partially by very old clinical studies of lower than usual quality. Clinical symptoms were PRO/ObsRO in one published study (primary endpoint) and PRO only in another (exploratory). No COAs reported from non-published studies. |
| trifarotene | Acne vulgaris | PRO/ClinRO: Local tolerability (S)  ClinROs: IGA (1+), PGA (2+) | IGA; PGA; worsening in local tolerability | - |
| ubrogepant | Migraine | *PROs: Pain freedom (1+); Absence of MBS (1+)* | *Freedom from headache pain and MBS* | PED table stated no PED included in submission, yet both assessment of pain and MBS is based on patients’ eDiaries assessing headache pain severity and absence or presence of migraine-associated symptoms → PRO  The C-SSRS (ClinRO) was part of the regular safety assessment |
| upadacitinib | Moderately to severely active rheumatoid arthritis | PROs: HAQ-DI (2+); FACIT-F (2+); morning stiffness (2+); SF-36 PCS (2+); SF-36 MCS (2*)  ClinROs: mTSS (2+); DAS28-CRP (2+)  PRO/ClinRO composite measure: ACR (1+) | ACR response; DAS28-CRP; mTSS  HAQ-DI; SF-36 (PCS and MCS); FACIT-F | Generally thorough discussion of the rationale for accepting inclusion of the individual COAs in the product labeling.  Not clear why “*the general improvement in morning stiffness is clinically useful and supports the primary endpoint that UPA improves the signs and symptoms of RA in a clinically meaningful manner*” did not translate into a label claim. |
| voxelotor | Sickle cell disease | PROs: EQ-5D-5L (E), SCDSM (E); school and/or work attendance as recorded in eDiary (E)  ClinRO: CGI-C (E) | - | The PROs were exploratory endpoints and no formal conclusions can be drawn from these results |
| zanubrutinib | Mantle cell lymphoma | - | - | No PED submitted as part of application as stated in PED table |

**Symbols:** (1) – COA formed the basis of a primary endpoint; (2) – COA formed the basis of a secondary endpoint; (E) – COA formed the basis of an exploratory endpoint: (2→E) – secondary endpoint reduced to exploratory status due to endpoints higher in testing hierarchy failing to reach statistical significance; (S) – COA formed the basis of a safety endpoint only (note: if a COA was included both for efficacy and safety assessment, only the efficacy status – e.g. E for exploratory – is listed); + – endpoint reached statistical significance (or was assessed as demonstrating convincing effect in case of single arm trials); ÷ – endpoint failed to reach statistical significance; * – mixed results (positive and negative, and/or inconclusive) or results not possible to interpret.

**Abbreviations:** 6MWT - 6 Minute Walking Test; AIMS - Abnormal Involuntary Movement Scale; ARR – Annualized Relapse Rate; BARS - Barnes Akathisia Rating Scale; BDI - Beck Depression Inventory; BPI - Brief Pain Inventory; BPI-Q - Brief Pain Inventory Questionnaire; BPI-SF – Brief Pain Inventory – Short Form; B/R - Benefit/Risk; BSS - Beck Scale for Suicidal ideation; CDSS - Calgary Depression Scale for Schizophrenia; CFQ-R RD - Cystic Fibrosis Questionnaire-Revised Respiratory Domain; CGI-C - Clinical Global Impression of Change; CGI-S - Clinical Global Impression–Severity; ClinRO – Clinician Reported Outcome; CRT - Choice Reaction Yime; CSBM - Complete Spontaneous Bowel Movement; C-SSRS - Columbia Suicide Severity Rating Scale; DAS28-CRP - Disease Activity Score in 28 joints using C-reactive protein level; DAT - Divided Attention Test; DLQI - Dermatology Life Quality Index; DSST - Digit Symbol Substitution Test; ECOG-PS - Eastern Cooperative Oncology Group (ECOG) Performance Status; ECR – Early Clinical Response; EDQ - Elements of Desire Questionnaire; EDSS - Expanded Disability Status Scale; EORTC - European Organization for the Research and Treatment of Cancer; EPP-QoL - Erythropoietic Protoporphyria QoL questionnaire; ESS - Epworth Sleepiness Scale; ETDRS - Early Treatment Diabetic Retinopathy Study; EQ-5D (or EuroQoL) - European Quality of Life Questionnaire; FACIT-F - Functional Assessment of Chronic Illness Therapy-Fatigue; FACT-MM - Functional Assessment of Cancer Therapy–Multiple Myeloma; FACT-P - Functional Assessment of Cancer Therapy – Prostate; FOSQ-10 - short version of the 30-item Functional Outcomes of Sleep Questionnaire; FSDS-DAO Q13 - Female Sexual Distress Scale – Desire/Arousal/Orgasm Question 13; FSEP-R Q10 - Female Sexual Encounter Profile – Revised, Question 10; FSFI-D Q1&2 - Female Sexual Function Index – Desire Domain Question 1 and 2; FSS - Fatigue Severity Scale; FVC - Forced Vital Capacity; GAIS - Global Aesthetic Improvement Scale; GAQ - General Assessment Questionnaire; GIC-QoL – Global Impression of Change – Quality of Life; GIC-S – Global Impression of Change – Symptoms; GLS - Glabelar Line Scale; HADS - Hospital Anxiety and Depression scale; HAQ-DI - Health Assessment Questionnaire-Disability Index; IACR - Investigator’s Assessment of Clinical Response; IBS-QOL – Irritable Bowel Syndrome-Quality of Life; IGA - Investigator Global Assessment; ISI - Insomnia Severity Index; KCCQ-OS - Kansas City Cardiomyopathy Questionnaire-Overall Summary; KSS - Karolinska Sleepiness Scale; LAD - Limited Activity Days survey; MBS - Most Bothersome Symptom; MCS - Mental Component Summary (of the SF-36); MoviPlate - a tool that measures the ability to produce repeated movements between 2 cylindrical target keys aligned in the sagittal plane; MSLT - Multiple Sleep Latency Test; MSWS - Multiple Sclerosis Walking Scale; mTSS - van der Heijde-modified Total Sharp Score; MWT - Maintenance of Wakefulness Test; NRS - Numeric Rating Scale; NSAA - North Star Ambulatory Assessment; OAA/S - Observer Assessment of Alertness/Sedation scale; OPAQ SV - Osteoporosis Assessment Questionnaire Short Version; PAB - Performance Assessment Battery; PANSS - Positive and Negative Syndrome Scale; PASI - Psoriasis Area and Severity Index; PCS - Physical Components Summary (of the SF-36); PDQ - Parkinson’s Disease Questionnaire; PerfO – Performance Outcome measure; PGA - Physician Global Assessment; PGI-C - Patient Global Impression of Change; PGI-I - Patient Global Impression – Insomnia; PODCI - Pediatric Outcomes Data Collection Instrument; PPEQ - Porphyria Patient Experience Questionnaire; PRO - Patient Reported Outcome; PROMIS - Patient-Reported Outcomes Measurement Information System; PSP - Personal and Social Performance Scale; PSQI - Pittsburgh Sleep Quality Index; PSS - Psoriasis Symptom Scale; PtGA - Patient Global Assessment; PUL - Performance Upper Limb; PVT - Psychomotor Vigilance Test; QLQ-C30 - Quality of Life Questionnaire; QLQ-LC13 - Quality of Life Questionnaire lung cancer module; QLQ-PR25 - Quality of Life Questionnaire – Prostate cancer module; QoL – Quality of Life; RTI - Reaction Time Index; SAS - Simpson-Angus Scale; SCDSM - The Sickle Cell Disease Severity measure; SDC - Symbol Digit Coding; SDLP - Standard Deviation of the mean Lateral Position; SF-12 PCS – Short Form-12, Physical Component Summary; SF36 - Short Form-36; SMQ - Symptom Measurement Questionnaire; sPGA - static Physician’s Global Assessment; SSE - Satisfying Sexual Events; SSTM - Sternberg Short-Term Memory; sSEF – Subjective Sleep Efficiency; sSOL - Subjective Sleep Onset Latency; SSS - Subject Satisfaction Scale; StSS – Stanford Sleepiness Scale; sTST – Subjective Total Sleep Time; sWASO - Subjective Wake After Sleep Onset; S-PSD - Self-Perceived Safety to Drive; SWN-S - Subjective Well-being under Neuroleptic treatment Scale; T25FW - 25-Foot Walk test; TINAS - Therapy-Induced Neuropathy Assessment Scale; TranQoL - Transfusion-dependent QoL questionnaire; TSMQ - Treatment Satisfaction Questionnaire for Medication; T-BWSQ - Tyrer Benzodiazepine Withdrawal Symptom Questionnaire; UPDRS - Unified Parkinson’s Disease Rating Scale; VAS – Visual Analog Scale; VAS-MSAD - Visual Analog Scale for Motivation and Self Appraisal for Driving VFQ-25 - Visual Function Questionnaire 25; WITS-9 - Women’s Inventory of Treatment Satisfaction; WOMAC - Western Ontario and McMaster Universities Arhritis Index; WPAI-GH – Work Productivity and Activity Impairment questionnaire; WPAI-SHP - Work Productivity and Activity Impairment Questionnaire: Specific Health Problem

1. The maintenance of wakefulness test (MWT) is an objective (based on a polysomnographic procedure) assessment of an individual’s ability to remain awake while resisting the pressure to fall asleep during soporific circumstances. The authors were uncertain of whether the MWT should be categorized as a COA (PerfO), but concluded by consensus that the MWT is a PerfO. [↑](#footnote-ref-2)
